# Evaluation of the cytochrome P450-mediated drug interaction profile of olorofim

**DOI:** 10.1128/aac.01745-25

**Published:** 2026-04-20

**Authors:** Karen Cornelissen, John H. Rex, Daniela Zinzi, Derek Law, Rachel Upcott Gill, Hannah M. Jones, Johan Maertens, Sharon C.-A. Chen, Roger Brüggemann

**Affiliations:** 1 F2G Ltd, Nether Alderley, Macclesfield, United Kingdom; 2 Cyprotex Discovery Ltd 380191 , Macclesfield, United Kingdom; 3 Certara UK Ltd 102555 , Sheffield, United Kingdom; 4 Department of Hematology, University Hospitals Leuven 60182 , Leuven, Belgium; 5 Centre for Infectious Diseases and Microbiology Laboratory Services, Department of Infectious Diseases, Institute for Clinical Pathology and Medical Research, New South Wales Health Pathology, Westmead Hospital, The University of Sydney 4334, Sydney, Australia; 6 Radboud University Medical Centre 6034, Nijmegen, the Netherlands; University Children's Hospital Münster, Münster, Germany

**Keywords:** drug interaction, olorofim

## Abstract

Olorofim is a novel oral antifungal agent which selectively inhibits the fungal enzyme dihydroorotate dehydrogenase found in molds including *Aspergillus*, *Scedosporium*, *Lomentospora*, and *Scopulariopsis* and in dimorphic fungi such as *Coccidioides*. Based on *in vitro* studies, clinical pharmacology, and pharmacokinetic (PK) data from the Phase 2b FORMULA study (F901318/0032) as well as physiologically based PK (PBPK) modeling of drug interactions, the drug’s CYP-mediated interactions are now well understood. Olorofim is mainly metabolized by cytochrome P450 (CYP) CYP3A4, with minor contributions from CYP2C8 and CYP2C9. *In vitro* studies found that it inhibits CYP3A4, weakly inhibits CYP2D6 and CYP2C8, and weakly induces CYP1A2 and CYP2B6. Phase I studies confirm it is predominantly cleared by CYP3A4 and weakly inhibits CYP3A4. In the Phase 2b FORMULA study, co-administered sensitive CYP3A4 substrates required limited dose reductions when using olorofim. When used with strong CYP3A4 inhibitors, dual moderate inhibitors like fluconazole, or CYP3A4 inducers, dose adjustments were needed for olorofim. Physiologically based PK (PBPK) modeling predicted moderate effects with CYP3A4 inhibitors or inducers and negligible effects with weak CYP3A4 inhibitors or strong inhibitors of CYP2D6, CYP2C8, and CYP2C9. Overall, the drug’s interaction profile is predictable and manageable, making it a suitable candidate drug to use in treating patients with invasive fungal disease (IFD), who often require polypharmacy.

**CLINICAL TRIALS:**

This study is registered with ClinicalTrials.gov as NCT02680808, NCT02730442, NCT02737371, NCT04171739, NCT03340597, and NCT03583164.

## INTRODUCTION

Invasive fungal diseases (IFDs) cause substantial morbidity and mortality, particularly in immunocompromised, individuals including those with neutropenia, transplant recipients, and other vulnerable patients with serious medical conditions (1).

Three classes of antifungals are currently approved: polyenes, azoles, and echinocandins, with only the azoles offering oral therapy options (1–3). However, the azoles are all either moderate or strong inhibitors of one or more CYP enzymes, which is a serious clinical concern for patients with IFD, who frequently receive numerous concomitant CYP450-dependent medications to manage underlying conditions (4). Given the limited treatment options available for an increasing number of IFD, expanding the antifungal armamentarium with an orally available agent which has a limited drug interaction profile is likely to be beneficial.

Olorofim is the first member of the orotomide class of antifungals and is a novel oral treatment of IFD due to mold fungi such as *Aspergillus* spp., *Scedosporium* spp., *Lomentospora prolificans*, *Scopulariopsis* spp., and *Coccidioides* spp. Olorofim inhibits fungal dihydroorotate dehydrogenase (DHODH), for which it is highly selective compared with the human form of the enzyme (5).

Olorofim is well absorbed following oral administration (6), with a single oral dose of 150 mg (using the currently available 30 mg tablet formulation) having a mean absolute bioavailability of 68% and linear pharmacokinetics (PKs) over the therapeutic dose range (60–150 mg bid). Olorofim is >99% protein bound, and the high apparent volume of distribution (mean *V*_ss_ of ~3 L/kg) is suggestive of extensive tissue distribution. The mean terminal half-life of 24–30 h, together with the presence of multiple peaks emerging during the elimination phase, results in plasma concentrations being maintained above the minimum threshold of 0.1 µg/mL needed for efficacy (7, 8); this level was exceeded throughout the 12 h dosing interval in over 99% of patients receiving olorofim in the Phase 2b FORMULA study (7, 8).

*In vitro*, olorofim was extensively metabolized by hepatocytes from the different species tested (mouse, rat, rabbit, minipig, dog, cynomolgus monkey, and human) across a wide concentration range. Further studies were designed to elucidate the cytochromes involved and the potential subsequent interactions with concomitant medications.

We summarize here the CYP-mediated drug interaction data available on olorofim, looking at olorofim as both victim and perpetrator of DDIs. Results have been combined from the *in vitro* setting, clinical pharmacology drug-drug interaction studies, drug interaction PK data from patients with IFD in the Phase 2b FORMULA study (Study F901318/0032), and physiologically based PK (PBPK) modeling.

## RESULTS

### Olorofim as perpetrator (CYP inhibitor and/or inducer): *in vitro* evaluation

#### CYP inhibition

Olorofim was evaluated *in vitro* as a perpetrator of DDIs due to metabolism mediated by CYP1A2, CYP2B6, CYP2C8, CYP2C9, CYP2C19, CYP2D6, and CYP3A4, using guidance-recommended approaches. In the assays conducted with human liver microsomes, the positive control inhibitors (listed in Table 1) behaved as expected, demonstrating assay suitability to evaluate olorofim as a CYP inhibitor. Olorofim IC_50_ values and fold-shift values (ratio of olorofim IC_50_ after 30 min incubation in the absence of NADPH to olorofim IC_50_ after 30 min incubation in the presence of NADPH) for each CYP isoform are given in Table 1. Olorofim did not inhibit CYP1A2, CYP2B6, CYP2C9, or CYP2C19 under any of the conditions tested; reversible inhibition was observed for CYP3A4, and the quantifiable fold-shift values for CYP3A4, CYP2D6, and CYP2C8 suggest that olorofim may exhibit time-dependent inhibition (TDI) of these enzymes. For CYP2D6 and CYP3A4, which showed quantifiable values for both IC_50_ and fold-shift, further *in vitro* assays were performed to define the inhibitory kinetic parameters for each isoform. Taking the olorofim IC_50_ values for each CYP enzyme into account and using estimated steady-state concentrations of total olorofim in the intestine and maximum unbound olorofim in plasma (to account for pre-systemic and systemic clearance) (9, 10), olorofim was predicted to only be a reversible inhibitor of CYP3A4. At therapeutically relevant concentrations, the potential for reversible CYP2D6 inhibition was not considered a risk. When looking at time-dependent inhibition parameters for CYP3A4 and CYP2D6, the data also show that there is a lesser risk that olorofim may inhibit CYP2D6 (Table 1).

**TABLE 1:** *In vitro* assessment of olorofim as a potential CYP inhibitor^*d*^

Isoform	Substrate [*Positive control*]	Incubation time post-substrate addition	Olorofim IC_50_ ± SE (µM)		Fold shift value	ICH DDI risk assessment^*a*^	
			0 min pre-incubation	30 min pre-incubation without NADPH [with NADPH]		Reversible DDI potential	TDI potential
CYP1A2	Phenacetin (18 µM) *[Furafylline (0.25 µM)]*	5 min	>25	>25.0 [>25.0]	None	Unlikely	Unlikely^*b*^
CYP2B6	Bupropion (150 µM) *[ThioTEPA (0.15 µM)]*	5 min	>25	>25.0 [>25.0]	None	Unlikely	Unlikely^*b*^
CYP2C8	Amodiaquine (2 µM) *[Gemfibrozil (0.1 µM)]*	5 min	>25	>25.0 [5.87 ± 1.71]	>4.26	Unlikely	ND^*c*^
CYP2C9	Diclofenac (7 µM) *[Tienilic acid (0.1 µM)]*	5 min	>25	20.5 ± 2.84 [>25.0]	None	Unlikely	Unlikely^*b*^
CYP2C19	Mephenytoin (50 µM) *[Fluoxetine (0.1 µM)]*	15 min	>25	>25.0 [>25.0]	None	Unlikely	Unlikely^*b*^
CYP2D6	Dextromethorphan (2 µM) *[Paroxetine (0.1 µM)]*	5 min	7.12 ± 1.56	6.00 ± 1.11 [1.15 ± 0.111]	5.21	Unlikely	Possible
CYP3A4	Midazolam (2.5 µM) *[Verapamil (0.25 µM)]*	5 min	9.70 ± 2.66	8.42 ± 2.48 [0.422 ± 0.0840]	19.9	Possible	Possible
CYP3A4	Testosterone (50 µM) *[Verapamil (0.25 µM)]*	5 min	3.07± 0.807	2.60 ± 0.552 [0.249 ± 0.0286]	10.5	Possible	Possible

^*a*^ Risk assessment as per ICH Harmonized Guideline: Drug Interaction Studies M12 Draft 24 May 2022.

^*b*^ Fold shift value indicative of no TDI potential.

^*c*^ Fold shift value suggestive of TDI potential; further evaluation to assess risk not performed.

^*d*^ DDI, drug-drug interaction; ND, not determined; SE, standard error; TDI, time-dependent inhibition.

#### CYP induction

Across the three donor human hepatocyte assays, the positive control inducers (listed in Table 2) behaved as expected, demonstrating assay suitability to evaluate olorofim as an inducer of CYP1A2, CYP2B6, and CYP3A4. A decreasing trend in mRNA levels was observed at higher olorofim concentrations in two donors for all three CYP enzymes assessed. For one of the three donors, olorofim caused concentration-dependent increases in mRNA expression of CYP1A2, CYP2B6, and CYP3A4, with maximum induction of 2.2-fold, 2.0-fold, and 2.63-fold, respectively. Where calculable, the level of induction observed with olorofim for this one donor was approximately 1% of that observed with the positive-control compounds, omeprazole, phenobarbital, and rifampicin (Table 2).

**TABLE 2:** *In vitro* assessment of olorofim as a potential CYP inducer^*d*^

CYP mRNA expression (Positive control)	Parameter	Donor 1	Donor 2	Donor 3
CYP1A2 (omeprazole)	Maximum observed fold induction (concentration)	NI	2.16***^b^ (4 µM)	NI
	% of positive control response	NI	0.7%	NI
CYP2B6 (phenobarbital)	Maximum observed fold induction (concentration)	NI	1.99**^a^ (0.4 µM)	NI
	% of positive control response	NI	NC^*c*^	NI
CYP3A4 (rifampicin and phenobarbital)	Maximum observed fold induction (concentration)	NI	2.63***^b^ (0.4 µM)	NI
	% of positive control response	NI	1.3%	NI

^*a*^ **P < 0.01, significantly different compared to vehicle control.

^*b*^ ***P < 0.001, significantly different compared to vehicle control.

^*c*^ NC, not calculated (maximum induction < 2-fold over vehicle control).

^*d*^ NI, No indication (statistically significant increase in mRNA expression compared to vehicle control not observed over the concentration range tested).

### Olorofim as perpetrator: clinical pharmacology studies

Based on the *in vitro* data, the most clinically significant CYP-mediated drug interaction was the potential for olorofim to inhibit CYP3A4. This drug interaction was therefore evaluated in man using midazolam as a sensitive CYP3A4 substrate.

**Fig 1:**
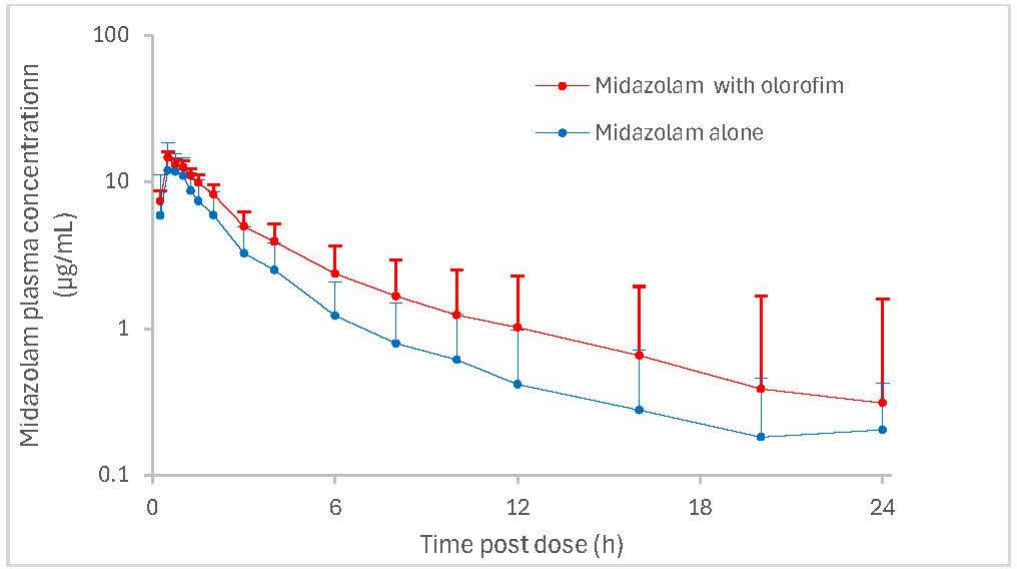
Mean (±SD) plasma concentrations of midazolam (semi-logarithmic scale) in the absence and presence of olorofim. Midazolam dose (oral) = 2 mg on Days 1 and 7, olorofim dose (IV) = 4 mg/kg twice daily on Day 3, 2.5 mg/kg twice daily on Days 4–6, and 2.5 mg/kg on Day 7.

Administering a single oral dose of midazolam (2 mg) in the presence of olorofim (maintenance dose of 2.5 mg/kg bid) to 20 healthy subjects resulted in a minimal increase in midazolam systemic exposure with *C*_max_ increasing by 1.20-fold (90% CI: 1.05–1.38) and AUC_0–*t*_ increasing by 1.54-fold (90% CI: 1.31–1.80, Fig. 1) (11). In accordance with regulatory guidance (10), the magnitude of impact categorizes olorofim as a weak CYP3A4 inhibitor.

### Olorofim as perpetrator: patients with IFD

Most of the patients in the Phase 2b FORMULA study (8) had underlying co-morbidities, including hematological malignancies, stem cell and solid organ transplants, who required treatment with chemotherapy agents and immunosuppressants, many of which are also sensitive substrates of CYP3A4. As Phase I data found olorofim to be a weak inhibitor of CYP3A4, prospective dose changes of sensitive CYP3A4 substrates were not recommended in the Phase 2b FORMULA study; Investigators were requested to adjust the dose of these concomitant medications based upon clinical signs of toxicity and/or TDM data.

Within 14 days of starting co-administration with olorofim, and where baseline dosing details were available, slight reductions in the dose of CYP3A4 substrates were required for 50% of evaluable patients receiving cyclosporine (9 out of 18 patients), where a median cyclosporine dose reduction of 50% was required (equating to a median numerical dose decrease of 47 mg [range: 25–100 mg]). For tacrolimus, 17% of evaluable patients (8 out of 46 patients) required a median tacrolimus dose reduction of 29% (equating to a median numerical dose decrease of 1 mg [range: 0.5–6.5 mg]). None of the three evaluable patients receiving sirolimus required a dose reduction within the 14-day period. In the other patients receiving these three medications, the dose either remained constant or was increased during co-administration with olorofim. A total of 6 patients were receiving tyrosine kinase inhibitors (bosutinib, ponatinib, dasatinib, and imatinib), and 10 were receiving venetoclax concomitantly with olorofim. For these patients, no dose reductions were needed within 14 days of starting treatment with olorofim, or if the concomitant medication commenced during the study, either a constant dose was administered or the recommended dose titration (e.g., as applied to venetoclax treatment) was successfully undertaken.

### Olorofim as perpetrator: PBPK modeling

Consistent with the non-clinical and clinical data, PBPK modeling predicted that olorofim is a weak inhibitor of CYP3A4 and CYP2D6, with 1.5-fold increases in exposures to the sensitive substrates midazolam and dextromethorphan, respectively, when co-administered with olorofim, compared to when dosed alone. As the model requires an estimate of the fraction of blood unbound in the gut, that is, within the enterocyte (fu_gut_; assumed to be equal to the fraction of olorofim unbound in plasma), even with a 10-fold increase in the fu_gut_ applied, the model predicts that the drug interaction would remain weak.

PBPK modeling assumed worst-case scenario for olorofim as a CYP1A2 and CYP2B6 inducer (using *in vitro* data from one liver donor; no induction was observed in the other two liver donors). A weak DDI was predicted, with AUC ratios (exposure of substrate + olorofim relative to exposure of substrate alone) of 0.5 for the CYP1A2 substrate (caffeine) and 0.7 for the CYP2B6 substrate (bupropion).

### Olorofim as victim (CYP substrate): *in vitro* data

#### CYP phenotyping

Olorofim was evaluated *in vitro* as a victim drug of CYP1A2, CYP2B6, CYP2C8, CYP2C9, CYP2C19, CYP2D6, and CYP3A4 using guidance-recommended approaches. Positive control compounds behaved as expected in the human microsome assays. Control incubations (incubation with microsomes in the absence of NADPH) demonstrated no chemical instability in the *in vitro* system used. Inhibition of CYP2C8, CYP2C9, and CYP3A4 reduced the intrinsic clearance of olorofim by >20%; inhibition of the remaining four isoforms resulted in intrinsic clearance varying by ±15% (Table S1).

Taking the relative contributions assigned to each isoform in the *in vitro* CYP phenotyping study performed in human liver microsomes and the *in vivo* abundance of the isoforms, CYP3A4 was the major isoform involved in CYP metabolism of olorofim, accounting for 71.2% of olorofim CYP-mediated clearance, with smaller contributions of approximately 18.6% and 7.7% assigned to CYP2C9 and CYP2C8, respectively. Contributions for CYP2C19, CYP2D6, CYP1A2, and CYP2B6 were found to individually account for <2% of olorofim CYP-mediated clearance.

### Olorofim as victim: *in vivo* clinical pharmacology studies

As a substrate of primarily CYP3A4, and to a lesser extent CYP2C9, clinical DDI studies were performed to determine whether inhibition or induction of these enzymes impacted systemic exposure of olorofim.

#### Interaction with a dual inhibitor of CYP2C9 and CYP3A4

**Fig 2:**
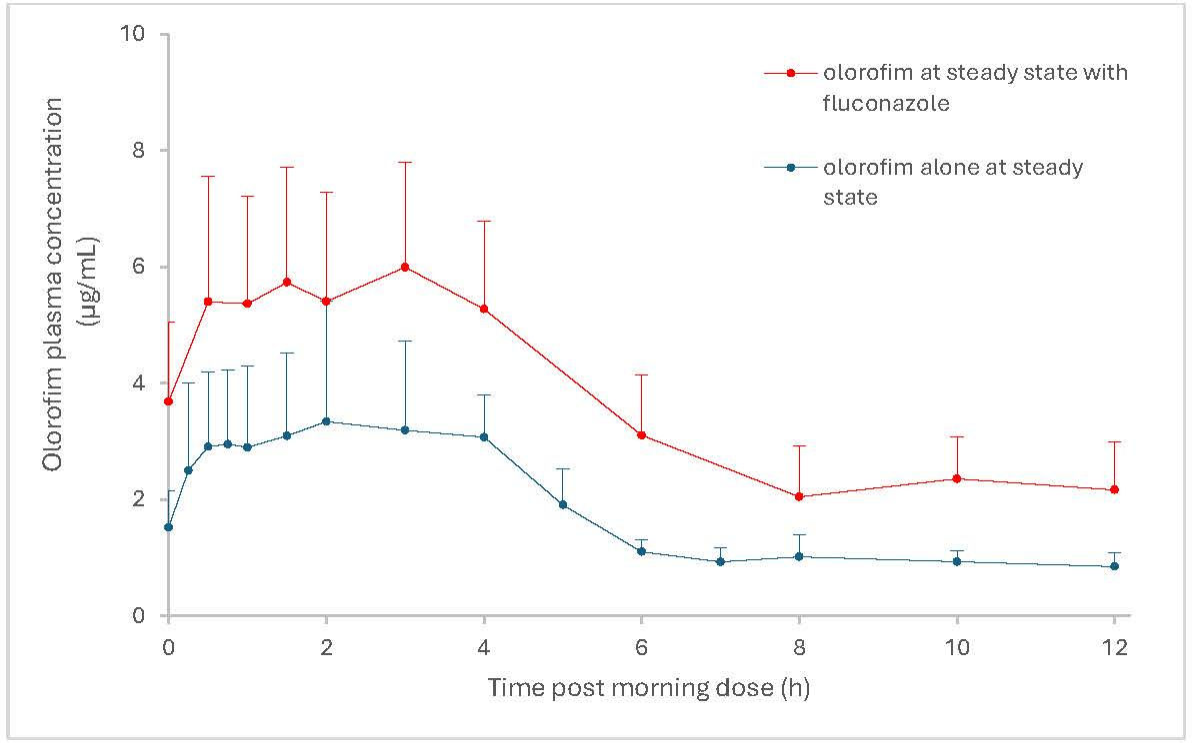
Mean (±SD) steady-state plasma concentrations of olorofim following multiple oral doses of olorofimgiven with and without fluconazole. Olorofim maintenance dose (oral): 2.5 mg/kg bid, fluconazole maintenance dose (oral) :400 mg once daily.

In the healthy volunteer study evaluating the effect of fluconazole (a dual moderate CYP3A4/CYP2C9 inhibitor; maintenance dose of 400 mg once daily) was found to have a modest impact upon a single IV dose of olorofim. Systemic exposure for olorofim (as assessed by AUC_0–72_) increased by 1.51-fold (90% CI: 1.39–1.64) and 1.56-fold (90% CI: 1.32–1.85) for 2 and 4 mg/kg olorofim, respectively, when given in the presence of fluconazole compared with when given alone. Upon repeat oral dosing of olorofim (maintenance dose of 2.5 mg/kg bid), fluconazole had a modest impact upon olorofim steady state exposure (12). Comparing steady-state exposure following administration of olorofim alone to that determined when olorofim was co-administered with fluconazole to a separate group of healthy volunteers (both studies using an olorofim maintenance dose of 240 mg bid), olorofim mean AUC_0–12_ and *C*_max_ increased by 2-fold and 1.7-fold, respectively when given together with fluconazole (Fig. 2).

#### Interaction with a strong CYP3A4 inhibitor

**Fig 3:**
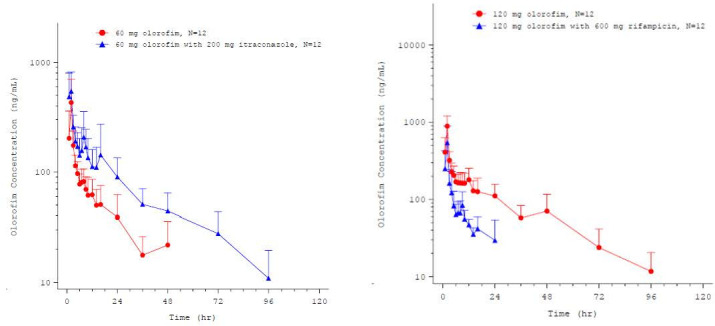
Mean (±SD) plasma concentrations of olorofim (semi-logarithmic scale) in the presence and absence of itraconazole (left-hand figure) and in the presence and absence of rifampicin (right-hand figure).

In the study evaluating the impact of a strong CYP3A4 inhibitor (itraconazole) upon the systemic exposure of single oral dose of olorofim (60 mg) in a single group of healthy volunteers, mean olorofim *C*_max_ and AUC_0–*t*_ increased when olorofim was co-administered with itraconazole (200 mg once daily) compared with when given alone (13) with geometric least square mean (LSM) ratios of 151.8% (90% CI: 117.7–196.0%) and 239.6% (90% CI: 200.3–286.7%), respectively (Fig. 3).

#### Interaction with a strong CYP3A4 inducer

When assessing the impact of a strong CYP inducer (rifampicin) in a single group of healthy volunteers, mean olorofim *C*_max_ and AUC_0–*t*_ decreased when a single oral dose of olorofim (120 mg) was co-administered with rifampicin (600 mg once daily) compared with when given alone, with geometric LSM ratios of 55.7% (90% CI: 41.5–74.9%) and 26.1% (90% CI: 22.5–30.3%), respectively (Fig. 3).

#### Impact of CYP polymorphism

**Fig 4:**
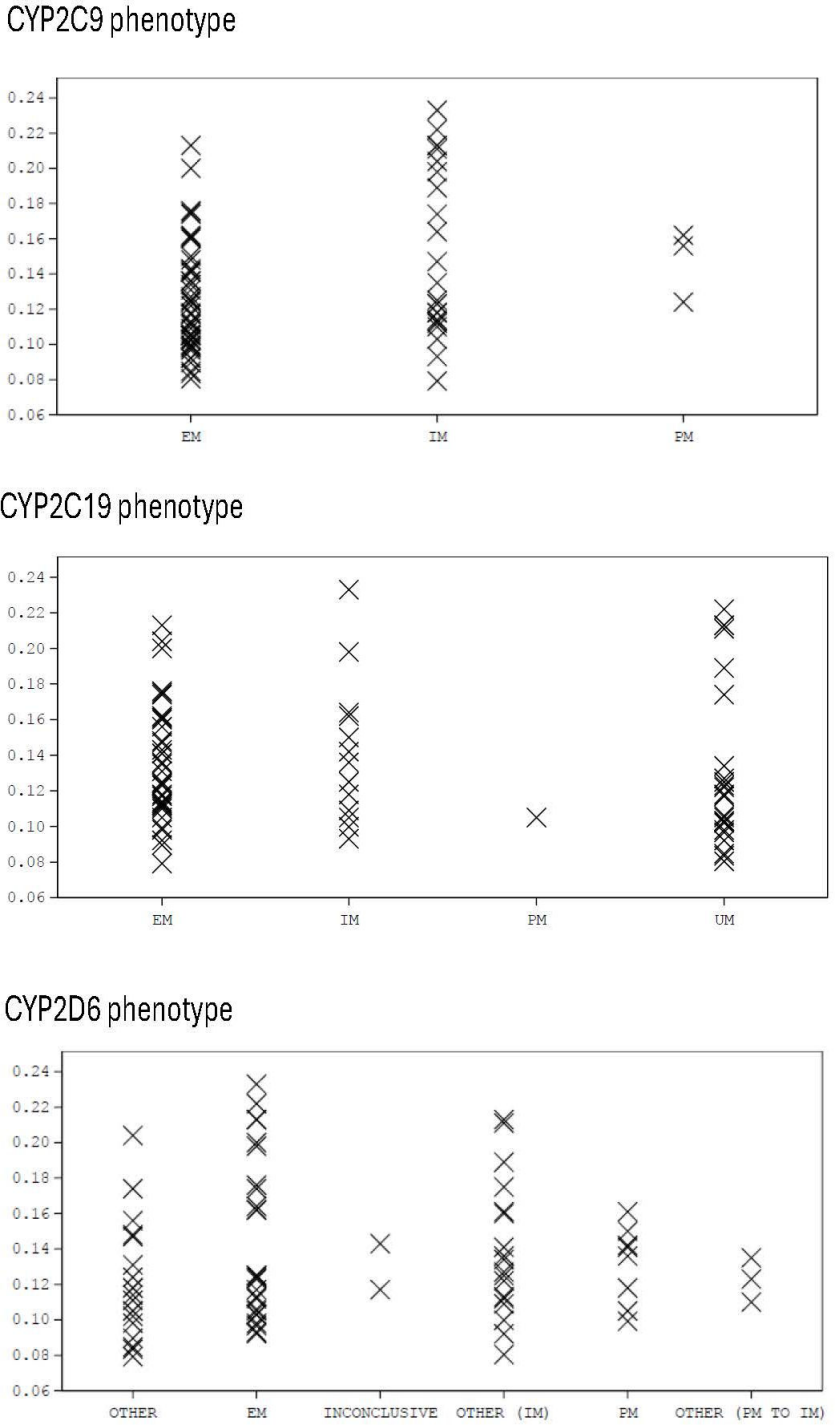
Systemic exposure of olorofim (as assessed by AUC/dose) by cytochrome P450 phenotype. EM, extensive metabolizer; IM, intermediate metabolizer; PM, poor metabolizer; UM, ultra metabolizer.

Due to the small contribution that the polymorphic CYPs (CYP2C9, CYP2C19, and CYP2D6) make to the overall clearance of olorofim, the systemic exposure of olorofim is not expected to be impacted by the CYP phenotype of the patient. This was confirmed in 45 healthy subjects receiving multiple oral doses of olorofim, where systemic exposure of olorofim for extensive metabolizers of CYP2C9, CYP2C19, and CYP2D6 was similar to that seen in intermediate or poor metabolizers of the three polymorphic CYPs or ultra-rapid metabolizers of CYP2C19 (Fig. 4).

### Olorofim as victim: Phase 2b data in patients with IFD

To further investigate the potential DDI profile for olorofim as a victim, olorofim systemic exposure was compared between patients in the Phase 2b FORMULA study who were receiving concomitant treatments that modified the activity of CYP2C9 and CYP3A4 and those who were not.

**Fig 5:**
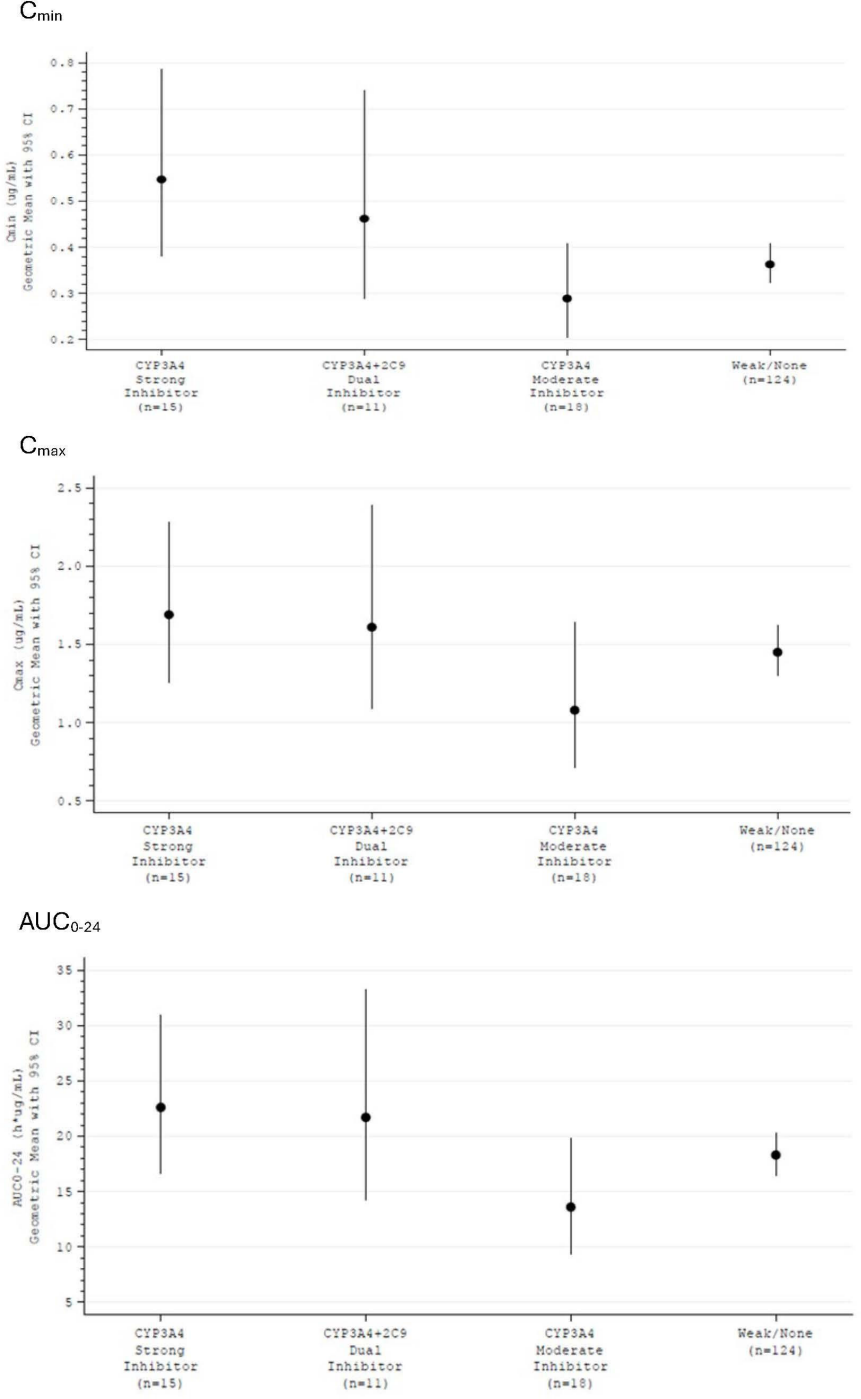
Systemic exposure of olorofim (A = *C*_min_; B = *C*_max_, and C = AUC_0–24_) in patients from the Phase 2b FORMULA study in the presence and absence of CYP3A4 inhibitors.

Overall, 19 patients received strong CYP3A4 inhibitors (posaconazole, voriconazole, itraconazole, clarithromycin, cobicistat, or ritonavir) concomitantly with olorofim, 17 additional patients were administered a dual moderate CYP3A4/CYP2C9 inhibitor (fluconazole), and a further 29 patients received moderate CYP3A4 inhibitors (isavuconazole, ciprofloxacin, letermovir, erythromycin, imatinib, diltiazem, or fluvoxamine). Looking at the subset of patients receiving both olorofim and a CYP inhibitor on the day when systemic exposure of olorofim was determined over a full dosing interval (16/19 [84%], 11/17 [65%], and 18/29 [62%] patients receiving strong CYP3A4 inhibitors, fluconazole, and moderate CYP3A4 inhibitors, respectively), the impact of co-administration was assessed. Figure 5 presents *C*_max_, *C*_min_, and AUC_0–24_ by CYP3A4 inhibitor category.

Systemic exposure for olorofim (as assessed by *C*_max_, *C*_min_, and AUC_0–24_) after a dose reduction of 30 mg/dosing occasion was similar in patients receiving concomitant strong CYP3A4 inhibitors or dual moderate CYP3A4/CYP2C9 inhibitors compared to patients receiving weak or no inhibitor (with no reduction in olorofim dose), with geometric mean AUC_0–24_ of 21.5 and 18.4 µg·h/mL, respectively, and corresponding *C*_max_ of 1.62 and 1.45 µg/mL and *C*_min_ of 0.522 and 0.364 µg/mL (see Fig. 5). These data suggest that the olorofim dose reduction of 30 mg per dosing occasion when olorofim is given concomitantly with these CYP inhibitors is appropriate (see Materials and Methods). Olorofim exposure was found to be slightly lower for patients receiving moderate CYP3A4 inhibitors (where the majority of patients [15/18] received a full dose of olorofim, i.e., no dose reduction was applied) compared to patients receiving full dose olorofim with weak or no CYP3A4 inhibitor, but the magnitude of change is of no clinical significance.

A further nine patients were administered a moderate CYP2C9 inhibitor (amiodarone, metronidazole, or miconazole) concurrently with olorofim. As per study guidance (which was based upon *in vitro* data), no prospective adjustment to olorofim dose was required when combined with CYP2C9 inhibitors. The average pre-dose plasma levels of olorofim in the three evaluable patients with pre-dose trough levels taken in the presence of a CYP2C9 inhibitor (range: 0.533–1.19 µg/mL) fell within the 5th–95th percentile range for the geometric mean across the remaining patients who did not receive any CYP inhibitors (0.21–2.35 µg/mL).

Four patients were administered a CYP inducer (carbamazepine and rifampicin) concurrently with olorofim. As per study guidance for increasing olorofim dose when co-administered with CYP inducers (up to a maximum of 150 mg bid, see Materials and Methods), the average pre-dose plasma levels of olorofim (range: 0.338–1.13 µg/mL) fell within the 5th–95th percentile range for the geometric mean across all remaining patients (0.25–2.15 µg/mL).

### Olorofim as victim: PBPK modeling

Interaction predictions from PBPK modeling were similar to those observed *in vitro* or in human studies. With regards to potential interactions between olorofim and CYP3A4 inhibitors and inducers, a moderate DDI was predicted with the strong CYP3A4 inhibitor itraconazole (AUC ratio of 2.62, range: 2.44–2.90), moderate CYP3A4 inhibitor verapamil (AUC ratio of 2.15; 90% CI: 2.04–2.27), strong CYP3A4 inducer rifampicin (AUC ratio of 0.24, range: 0.19–0.26) and moderate CYP3A4 inducer efavirenz (AUC ratio of 0.385; 90% CI: 0.361–0.411). A negligible DDI was predicted with the weak CYP3A4 inhibitor cimetidine.

For the remaining CYPs involved in olorofim clearance, negligible DDIs were predicted between olorofim and the strong CYP2D6 and CYP2C8 inhibitors, quinidine and gemfibrozil, respectively. In the absence of an FDA/EMA recommended strong CYP2C9 inhibitor, a complete knockout of CYP2C9 activity was found to lead to a weak DDI with olorofim.

## DISCUSSION

*In vitro* data found that olorofim is an inhibitor of CYP3A4, CYP2D6, and CYP2C8. It is also a weak inducer of CYP1A2 and CYP2B6 (see Table 3). Clinical DDI studies conducted with healthy volunteers have shown that, for CYP3A4, the *in vitro* data translate into man: olorofim is a weak inhibitor of CYP3A4, with a 1.2-fold increase in *C*_max_ and 1.5-fold increase in AUC of the sensitive CYP3A4 probe substrate midazolam. The clinical significance of olorofim impacting CYP2C8, CYP2D6, CYP1A2, and CYP2B6 is less clear. However, the interaction is likely to be weaker than that observed for CYP3A4 as the *in vitro* inhibition parameters determined for CYP2D6 and CYP2C8 (IC_50_ and/or fold-shift increase) were less than those observed for olorofim’s inhibition of CYP3A4 (see Table 1) and induction of CYP1A2 and CYP2B6 was only seen in hepatocytes from one out of three donor livers assessed where the mRNA increase produced by olorofim was approximately 100-fold less than that produced by the positive controls (see Table 2).

**TABLE 3:** Clinically demonstrated CYP-mediated drug interaction potential of olorofim and the commercially available azoles^*a*^^,^^*b*^^,^^*c*^^,^^*e*^^,^^*f*^

	Olorofim	Fluconazole	Isavuconazole	Itraconazole	Posaconazole	Voriconazole
Antifungal agent as an inhibitor (perpetrator)						
CYP2C9		**++**		**^+^**		**++**
CYP2C19		**+++**				**++**
CYP2D6						
CYP3A4	**+**	**++**	**++**	**+++**	**+++**	**+++**
Antifungal agent as a sensitive substrate (victim)						
CYP2C9						**+**
CYP2C19						**+++** ^*d*^
CYP3A4	**+**		**+++**	**+++**		**+**

^*a*^ +, Weak DDI (confirmed clinically: AUC increased by 1.25 to <2-fold).

^*b*^ ++, Moderate DDI (confirmed clinically: AUC increased by ≥2-fold to ≤5-fold).

^*c*^ +++, Strong DDI (confirmed clinically: AUC increased by >5-fold).

^*d*^ Clinically relevant increase in exposure seen in poor metabolizers.

^*e*^ *In vitro* data alone suggest that olorofim may be a weak inhibitor of CYP2D6 and CYP2C8, may be a weak inducer of CYP1A2 and CYP2B6, and may be a weak substrate of CYP2C9.

^*f*^ Data for the azoles are from prior reports reports (14, 15).

Triazoles represent the standard of care for antifungal treatment, but they have a complex DDI profile; all are either moderate or strong CYP3A4 inhibitors, and some (fluconazole, itraconazole, and voriconazole) also inhibit CYP2C9 and CYP2C19 (see Table 3). As a consequence, azoles are co-administered with caution with many other agents. Particularly, coadministration of azoles with CYP3A4 substrates with a narrow therapeutic window will generate concern in achieving therapeutic concentrations whilst avoiding systemic exposures which are associated with toxicity. In contrast, olorofim, as a weak CYP inhibitor, has a more manageable profile (see Table 3), where the PBPK model built using both *in vitro* data and *in vivo* DDI data drove the recommendation for no dose adjustment for CYP3A4 substrates when taken concomitantly with olorofim. This is confirmed by data from the Phase 2b FORMULA study (8), where looking at sensitive CYP3A4 substrates with narrow therapeutic windows such as the immunosuppressants cyclosporine and tacrolimus, only a subset of patients (50% and 17%, respectively) required a slight dose reduction of these concomitant medications upon starting treatment with olorofim. In contrast, all azoles have been shown to markedly increase the systemic exposure of the immunosuppressants cyclosporine, sirolimus, and tacrolimus in healthy subjects and/or patients with an underlying disease and can result in contraindications (e.g., sirolimus and voriconazole) (14). Other examples of sensitive CY3A4 substrates include hemolytic malignancy treatments such as tyrosine kinase inhibitors and venetoclax, which exhibit a greater risk of treatment-limiting drug toxicities if there is no dose reduction when co-administered with azoles (4). Such complexities appear to be avoided with olorofim, where based upon clinical assessments of toxicity, no dose changes were required in the 16 patients receiving such treatment. As olorofim has been shown to be a weak CYP3A4 inhibitor, and based upon the findings from patients with IFD and PBPK modeling, no prospective dose change in sensitive CYP3A4 substrates is required upon starting co-administration with this novel antifungal; dose reduction of the concomitant medication should be based upon clinical data such as adverse signs or symptoms or TDM data being outside target range.

Olorofim has been shown to be predominantly cleared by CYP-mediated metabolism. *In vitro* data found that CYP3A4 was the major clearance route and clinical pharmacology data demonstrated that olorofim systemic exposure is moderately impacted (defined as exposure being increased by 2- to 5-fold or decreased by 50%–80%) when co-administered with strong CYP3A4 inhibitors, the dual moderate CYP3A4/CYP2C9 inhibitor, fluconazole, and strong CYP3A4 inducers. Taking these data, the PBPK model predicted olorofim to be a victim of DDIs arising from inhibition and induction of CYP3A4 alone (negligible or weak DDIs were predicted for the other CYPs involved in olorofim clearance), which is in line with CYP3A4 fraction metabolized of approximately 70%. As a result, dose adjustment recommendations for olorofim were generated: decrease the dose by 30 mg per dosing occasion when co-administering with strong CYP3A4 inhibitors and fluconazole; increase the dose in a stepwise manner by 30 mg per dosing occasion up to a maximum of 150 mg bid when co-administering with either moderate or strong CYP3A4 inducers (see Table 4). Review of the data from the Phase 2b FORMULA study (8) is generally supportive of these dose adjustments. After the implementation of the study guidance on dose changes, olorofim systemic exposure was similar to that in patients who had not received CYP inhibitors or inducers. While subsets of evaluable patients were relatively small (3–4 patients with PK data receiving either moderate CYP2C9 inhibitors or CYP3A4 inducers and 11–18 patients with PK data receiving moderate or strong CYP inhibitors or fluconazole), the systemic exposures observed were in line with PBPK predicted DDI.

**TABLE 4:** Olorofim dose modification recommendations applied to Phase 2b FORMULA study

Cytochrome P450-mediated interaction	Olorofim dose modifications used
Strong inhibitors of CYP3A4 (e.g., itraconazole, posaconazole, voriconazole)	Reduce olorofim dose by 30 mg (one tablet) per dosing occasion throughout period of concomitant administration (e.g., reduce from 90 to 60 mg BID)
Dual moderate inhibitor of CYP2C9 and 3A4 (e.g., fluconazole)	Reduce olorofim dose by 30 mg (one tablet) per dosing occasion throughout period of concomitant administration (e.g., reduce from 90 to 60 mg BID)
Moderate inhibitor of CYP3A4 (e.g., isavuconazole)	No change to olorofim dose required
Weak inhibitors of CYP3A4	No change to olorofim dose required
Strong inducer of CYP3A4 (e.g., rifampin)	Increase olorofim dose by 30 mg (one tablet) per dosing occasion in a step-wise manner to a maximum of 150 mg BID, reverting back to standard olorofim dose 5–7 days after stopping treatment with the inducer (e.g., increase from 90 to 120 mg BID then after 2 weeks increase to 150 mg BID)
Moderate inducer of CYP3A4 (e.g., phenobarbital)	Increase olorofim dose by 30 mg (one tablet) per dosing occasion in a step-wise manner to a maximum of 150 mg BID, reverting back to standard olorofim dose 5–7 days after stopping treatment with the inducer (e.g., increase from 90 to 120 mg BID then after 2 weeks increase to 150 mg BID)
Weak inducer of CYP3A4	No change to olorofim dose required

Olorofim has been shown to be efficacious in patients with IFD who had few or no other treatment options, the majority of whom (125/202, 62%) received olorofim as monotherapy, with a further 18 (9%) receiving antifungal therapy for a limited duration (2–7 days), which is suggestive that concomitant administration with mold-active azoles is not necessary (8). If, however, co-administration with azoles (including fluconazole) is required (such as for the management of yeast infections), the clinical data collected to date suggest that such concomitant dosing can occur providing the olorofim dose changes used in the Phase 2b FORMULA study (see Table 4) are implemented.

The impact of olorofim’s DDI potential with specific subsets of the populations can also be estimated based upon data generated to date. As olorofim systemic exposure is not impacted by mild or moderate hepatic impairment (16), or by poor metabolizers or ultra metabolizers of the polymorphic CYPs (CYP2C9, CYP2C19, and CYP2D6), the magnitude of DDI is not predicted to be impacted by hepatic impairment or CYP polymorphism. This is in contrast to voriconazole where, if the dose is not prospectively adjusted, the magnitude of DDI may increase in patients with mild or moderate hepatic impairment, where exposure increases approximately 2-fold, or in poor metabolizers of CYP2C19, where systemic exposure is approximately three to five times higher than in extensive metabolizers (17, 18), and with notable ethnic differences in frequencies (e.g., 3% of Caucasians are poor metabolizers of voriconazole compared to 14–19% of Asians (19).

In summary, the DDI profile of olorofim both as a perpetrator and victim has been shown to be clinically manageable. In contrast to azoles, the gold standard approved class of orally administered antifungal treatments, olorofim is a weak inhibitor of CYP3A4, whereas each azole is at least a moderate inhibitor of one or more CYPs (14, 15, 20). In addition, olorofim is only a moderately sensitive substrate of CYP3A4, whereas three of the azoles are sensitive substrates of a CYP enzyme (Table 3) (15, 21, 22). In the setting of IFD treatment where most patients have underlying disease and consequently are subject to polypharmacy, having to modify the dosage of existing treatments or switch to an alternative treatment when starting with an azole adds complexity and risk to the treatment plan of the patient. As such, olorofim appears to offer a clinically manageable drug interaction profile and can be administered in conjunction with sensitive CYP3A4 substrates with narrow therapeutic windows including most immunosuppressive agents or hematologic malignancy treatments without prospective empirical dose adjustment.

## MATERIALS AND METHODS

### *In vitro* assays

All *in vitro* assays evaluating whether olorofim is a substrate, inhibitor, or inducer of CYP enzymes were setup in line with the current ICH/FDA/EMA guidelines and conducted at a single facility (Cyprotex, Macclesfield) in accordance with their standard operating procedures and in-house methodologies. All consumables were purchased from reputable suppliers.

#### CYP inhibition

The potential for olorofim to act as an inhibitor of a range of CYP isoforms was investigated *in vitro*. Olorofim (0.1, 0.25, 1, 2.5, 10, and 25 μM) was subject to 0 or 30 min pre-incubation with human liver microsomes (in the absence and presence of β-nicotinamide adenine dinucleotide phosphate, reduced [NADPH]), after which NADPH and an appropriate isoform-specific probe substrate at *K*_m_ (as shown in Table S1) was added and incubated for a specific time as defined for each isoform. Formation of substrate-specific metabolites was monitored, metabolite peak area in the presence of test compound was expressed as a percentage of metabolite peak area for vehicle control, plotted against nominal olorofim concentrations and fitted using a simple inhibitory model (WinNonLin model 103) to provide IC_50_ values. To assess time-dependent inhibition for those CYP isoforms that showed quantifiable values for both IC_50_ (concentration needed to inhibit CYP enzyme activity by 50%) and fold-shift (the magnitude of change in IC_50_ values from 30 min pre-incubation in the absence of NADPH to 30 min pre-incubation in the presence of NADPH), *k*_inact_/*K*_I_ assays were also performed to define the inhibitory kinetic parameters for each isoform. In the *k*_inact_/*K*_I_ assays, olorofim was pre-incubated with human liver microsomes in the presence of NADPH over a range of pre-incubation times. Following the pre-incubations, a dilution of the pre-incubation with NADPH and the isoform-specific substrate at 5 × *K*_m_ was performed and incubated for a specific time (relevant to the CYP isoform tested). Non-linear regression analysis of the *k*_obs_ values plotted against olorofim concentration was used to define *k*_inact_ and *K*_I_ values. To calculate *k*_obs_ values, the negative slope of the natural logarithm of the corrected % activity plotted against pre-incubation time was calculated.

#### CYP induction

The potential of olorofim to be an inducer of CYP isoforms CYP1A2, CYP2B6, and CYP3A4 was investigated in cryopreserved human hepatocytes in three individual donors. Olorofim was incubated for 72 h (media and drug replenished every 24 h). Following the incubation, cells were lysed, and RT-qPCR was performed. Samples were analyzed using an Applied Biosystems QuantStudio 7 Real-Time PCR system, and relative fold messenger ribonucleic acid (mRNA) expression was determined using the 2^−∆∆CT^ method (23).

#### CYP-mediated clearance

In the CYP phenotyping study, olorofim was incubated with human liver microsomes and NADPH for up to 45 min in the absence and presence of specific CYP inhibitors (Table S1). Negative controls (with inhibitor but without NADPH) and positive controls (compounds known to be specifically cleared by the CYP isoform being tested) were also assessed. Olorofim and positive controls were pre-incubated with the microsomes and P450-specific inhibitor for 5 min prior to the addition of NADPH to initiate the reaction. Following LC-MS/MS analysis of olorofim/positive controls, a semi-log plot of peak area ratio (olorofim peak area/internal standard peak area) against time was used for the calculation of intrinsic clearance (CL_int_).

### Clinical data

In all clinical trials, plasma concentrations of total olorofim were measured at a central bioanalytical laboratory (Resolian, UK) using a validated LC-MS/MS method.

#### Clinical pharmacology studies

The drug interaction profile of olorofim was evaluated *in vivo* in three clinical Phase 1 clinical pharmacology studies conducted in healthy subjects as enumerated below. These studies determined the impact of olorofim on CYP substrates PK (where olorofim is potentially the perpetrator) and looked at the effect of CYP inhibitors and inducers on olorofim PK (where olorofim is potentially the victim). Supratherapeutic olorofim doses of 2–4 mg/kg (140–290 mg for a 70 kg subject) were evaluated in two studies, with more therapeutically relevant single oral doses of 60 and 120 mg assessed in the third study (the maintenance dose used in Phase 2 and Phase 3 trials of olorofim being 90 mg bid).

#### Olorofim as a potential CYP inhibitor

To evaluate whether olorofim had the potential to inhibit CYP3A4, a clinical study in 20 healthy male subjects was performed to assess the impact of olorofim upon CYP3A4 (Study F901318-010415; clintrials.gov NCT02680808) (11).

Each subject received a single oral dose of midazolam (2 mg) on Day 1, supratherapeutic 4-h IV infusions of olorofim on Days 3– 6 (4 mg/kg bid on Day 3; 2.5 mg/kg bid thereafter), and on Day 7 a single oral dose of midazolam (2 mg) with a single olorofim infusion (2.5 mg/kg).

#### Olorofim as a victim of CYP inhibitors and inducers

Based on the relative contribution of CYP3A4 and CYP2C9 in the clearance of olorofim, a drug interaction study was performed in healthy volunteers with fluconazole (dual moderate CYP3A4/CYP2C9 inhibitor) (Study F901318-01-05-15; clintrials.gov NCT02730442) (12). In Part 1 of this drug-drug interaction (DDI) study, 20 healthy male subjects received single supratherapeutic IV doses of olorofim (2 or 4 mg/kg) on Days 1 and 8, with oral fluconazole being given on Days 4–8 (Day 4: 800 mg; Days 5–8: 400 mg QD [once daily]). PK sampling for olorofim occurred for 72 h after each olorofim dose. In Part 2, a total of 12 male subjects received oral olorofim and oral fluconazole concomitantly (Day 1: 4 mg olorofim/kg bid + 800 mg fluconazole; Days 2–4: 2.5 mg/kg olorofim bid + 400 mg fluconazole QD; and Day 5: 2.5 mg/kg olorofim + 400 mg fluconazole). PK sampling for olorofim occurred for 12 h post-dose on Days 1 and 5. The comparator group comprised seven healthy subjects (three male and four female) who received multiple oral doses of olorofim alone (4 mg/kg on Day 1, 2.5 mg/kg on Days 2–10), with PK sampling for olorofim for 12 h post-dose on Days 1 and 10 (Study F901318-01-06-16; clintrials.gov NCT02737371).

A further DDI study was performed in healthy volunteers to determine the impact of a strong CYP3A4 inhibitor, itraconazole, and a strong CYP3A4 inducer, rifampicin, on the systemic exposure of olorofim in healthy volunteers (Study F9013180115; clintrials.gov NCT04171739) (13). The study comprised two cohorts of 12 healthy male and female subjects. In Cohort A, each subject received a single oral dose of olorofim (60 mg) on Days 1 and 11, with doses of itraconazole oral solution (200 mg QD) administered on Days 6 –15. In Cohort B, each subject received a single oral dose of olorofim (120 mg) on Days 1 and 11, with oral doses of rifampicin (600 mg QD) administered on Days 6–15. PK sampling for olorofim occurred for 120 h after each olorofim dose.

#### Olorofim exposure in differing CYP phenotypes

A multiple dose study was performed looking at varying oral dosing regimens in healthy volunteers (Study number F901318-01-13; clintrials.gov NCT03340597). A total of 43 subjects received olorofim, with loading doses given on Day 1 and maintenance doses of 120–240 mg/day given for 9 days. A blood sample was collected prior to the first dose of olorofim and genotyping for CYP2C9, CYP2C19, and CYP2D6 was performed, from which the phenotype of each isoform was derived.

#### Drug interaction evaluations in patients with IFD

In Study 32, the Phase 2b FORMULA study of patients with IFD (8) NCT03583164, the PK profile of olorofim was determined and potential risk of DDIs when co-administering olorofim with specific CYP substrates, inhibitors, or inducers was assessed. The first 58 patients received an olorofim oral dose that was adjusted for body weight and olorofim pre-dose trough concentrations, with the remaining 145 patients receiving olorofim as a fixed oral dose regimen comprising a loading dose of 150 mg bid for 1 day followed by a maintenance dose of 90 mg bid for at least 84 days. Pre-dose trough levels of olorofim were determined on 8–10 occasions across the 84-day dosing period, and 6–8 plasma samples were taken over at least one dosing interval to determine olorofim minimum, maximum, and total exposure at steady state (*C*_min_, *C*_max_, and AUC_tau_, respectively). Systemic exposure was shown to be the same for the adjusted dose and fixed dose regimens; therefore, 203 patients are considered as a whole regardless of dosing regimen.

Assessment of the concomitant medications taken by patients enrolled in the Phase 2b study showed that numerous sensitive CYP3A4 substrates were taken. The impact of olorofim on these substrates has been assessed by looking at the dose changes required when starting olorofim treatment.

As *in vitro* and clinical pharmacology data suggest that olorofim is primarily cleared by CYP3A4 and to a lesser extent CYP2C9, dose modifications to olorofim when co-administered with specific CYP inhibitors and inducers were implemented in the Phase 2b study. While taking either a strong CYP3A4 inhibitor or fluconazole with olorofim, a 30–50% dose reduction was requested for all patients, where the dose was reduced by a single 30 mg tablet per dosing occasion. When a CYP inducer was administered concomitantly, a 50–100% dose increase was required (with the dose increased by a single 30 mg tablet for 2 weeks and if no adverse effects were noted, a further dose increase by an additional 30 mg tablet was permitted, up to a maximum of 300 mg /day). In addition, for the first 58 patients enrolled into the Phase 2b FORMULA study, a 30–50% reduction in olorofim dose was also required when receiving moderate CYP3A4 inhibitors, although upon review of emerging PK data, this was changed to no olorofim dose adjustment being needed. To assess the impact of CYP3A4 inhibitors and inducers upon olorofim exposure (after dose adjustment where required), summary statistics of the olorofim PK parameters *C*_min_, *C*_max_, and AUC_tau_ were compared between patients receiving CYP3A4 modifying agents to those not.

The demographics of study subjects across the three Clinical Pharmacology DDI studies and the Phase 2b FORMULA study are summarized in Table S2.

### PBPK model

A physiologically based pharmacokinetic (PBPK) model for olorofim was developed to predict the DDI risk for a single oral dose of 120 mg olorofim as a victim of DDIs mediated by inhibition and induction of CYP3A4 and inhibition of CYP2C9, CYP2C8, CYP2C19, and CYP2D6. The final model was also applied to predict the liability of multiple oral doses of 90 mg bid olorofim as an inducer of CYP1A2 and CYP2B6 and as an inhibitor of CYP3A4 and CYP2D6. The PBPK model was developed using the Simcyp Simulator version 21.

The PBPK model for olorofim was based on both *in vitro* data (including reversible and time-dependent inhibition parameters where available) and *in vivo* data and considered metabolism by a range of CYP isoforms. The model was developed using data from several clinical pharmacology studies conducted in healthy volunteers. Single intravenous doses were used to establish overall clearance and volume of distribution, and the drug interaction data between a single oral dose of midazolam and multiple intravenous infusions of olorofim were used to refine time-dependent inhibition of CYP3A4. The human absorption, metabolism, and excretion data were used to confirm that oxidative CYP-mediated metabolism was the major pathway of olorofim clearance, together with the relative contributions assigned from reaction phenotyping in recombinant human CYP (rhCYP) experiments. For the model development and verification simulations, outputs were based upon 10 virtual trials each of the same number of subjects as the clinical trial. For application simulations, outputs were based upon 10 virtual trials each of 10 subjects. Model input parameters are summarized in Table S3.

As part of model verification, the model demonstrated accurate independent recovery of observed plasma olorofim concentrations from several single-dose clinical studies, suggesting a good recovery of olorofim bioavailability and fraction absorbed. It has been indicated that for comparisons of predicted *versus* observed exposure of drugs, ratios within 0.5- to 2-fold (i.e., within 2-fold error) is “a primary metric for assessment of model fidelity” (24). For the modeling of olorofim, a more stringent approach was used where the recovery was defined as good when simulated summary PK parameters were within 0.80- to 1.25-fold of corresponding observed values. Good model recovery of olorofim PK after multiple dosing was also determined, demonstrating correct assignment of CYP3A4 auto-inhibition and steady-state PK. The model also showed a good recovery of the observed olorofim AUC and *C*_max_ ratios when dosed with the strong CYP3A4 inhibitor, itraconazole, the strong CYP3A4 inducer rifampicin, and the strong CYP2C19, moderate CYP3A4 and CYP2C9 inhibitor fluconazole. Key model verification results are summarized in Table S4. The good recovery of these DDI studies suggests that the assignment on the basis of *in vitro* rhCYP data of olorofim fraction metabolized for CYP3A4 and CYP2C9 of 71.7% and 18.6% of olorofim clearance, respectively (calculated from recombinant CYP data accounting for inbuilt inter-system extrapolation factors within the Simcyp Simulator and the abundance of each individual CYP in the human liver) was correct.

The verified olorofim PBPK model was applied to predict the liability of a single oral dose of 120 mg olorofim as a victim of DDIs when co-administered with a series of CYP inhibitors and inducers.

Olorofim’s ability to inhibit or induce CYP enzymes was classified per guidance (10) with strong, moderate, and weak inhibitors defined as drugs that increase the AUC of sensitive index substrates of a given metabolic pathway by ≥5-fold, ≥2- to <5- fold , and ≥1.25- to < 2-fold , respectively and strong, moderate, or weak inducers defined as drugs that decrease the AUC of sensitive substrates by ≥80%, ≥50% to <80% or ≥20% to <50%, respectively. When evaluating olorofim as a CYP3A4 inhibitor specifically, the application of CYP3A4 time-dependent inhibition from the *in vitro* kinact and KI parameters resulted in significant over-prediction of the weak DDI observed *in vivo* between olorofim and the CYP3A4 substrate midazolam. Therefore, a fourfold reduction of the *in vitro* kinact value was determined via sensitivity to allow the model to accurately reproduce the fold change in the midazolam *C*_max_ and AUC_0–24_. An over-prediction of CYP3A4-mediated DDIs when *in vitro* time-dependent inhibition parameters are directly applied in PBPK models is not uncommon and has previously been reported in the literature. For example, a 10-fold reduction in the *in vitro* CYP3A4 kinact was successfully applied in the simulation of CYP3A4 time-dependent inhibition by clarithromycin (25).

## SUPPLEMENTAL MATERIAL

Supplemental material aac.01745-25-s0001.docx Tables S1 to S4.

ASM does not own the copyrights to Supplemental Material that may be linked to, or accessed through, an article. The authors have granted ASM a non-exclusive, world-wide license to publish the Supplemental Material files. Please contact the corresponding author directly for reuse.

## References

[1] Sanguinetti M, Posteraro B, Beigelman-Aubry C, Lamoth F, Dunet V, Slavin M, Richardson MD. 2019. Diagnosis and treatment of invasive fungal infections: looking ahead. J Antimicrob Chemother 74:ii27–ii37. doi:10.1093/jac/dkz041 31222314

[2] Wall G, Lopez-Ribot JL. 2020. Current antimycotics, new prospects, and future approaches to antifungal therapy. Antibiotics (Basel) 9:445. doi:10.3390/antibiotics9080445 32722455 PMC7460292

[3] Hoenigl M, Salmanton-García J, Walsh TJ, Nucci M, Neoh CF, Jenks JD, Lackner M, Sprute R, Al-Hatmi AMS, Bassetti M, et al.. 2021. Global guideline for the diagnosis and management of rare mould infections: an initiative of the European Confederation of Medical Mycology in cooperation with the International Society for Human and Animal Mycology and the American Society for Microbiology. Lancet Infect Dis 21:e246–e257. doi:10.1016/S1473-3099(20)30784-2 33606997

[4] Brüggemann RJ, Verheggen R, Boerrigter E, Stanzani M, Verweij PE, Blijlevens NMA, Lewis RE. 2022. Management of drug-drug interactions of targeted therapies for haematological malignancies and triazole antifungal drugs. Lancet Haematol 9:e58–e72. doi:10.1016/S2352-3026(21)00232-5 34890539

[5] Oliver JD, Sibley GEM, Beckmann N, Dobb KS, Slater MJ, McEntee L, du Pré S, Livermore J, Bromley MJ, Wiederhold NP, Hope WW, Kennedy AJ, Law D, Birch M. 2016. F901318 represents a novel class of antifungal drug that inhibits dihydroorotate dehydrogenase. Proc Natl Acad Sci USA 113:12809–12814. doi:10.1073/pnas.1608304113 27791100 PMC5111691

[6] Cornelissen K, Newell PA, Rex JH, Galbraith H, Israel S, Bush J, ChB MB. 2022. 594. An open-label study in healthy volunteers to determine the absolute bioavailability of, the effect of food and dosing by nasogastric tube upon the pharmacokinetics of a single oral dose of olorofim (OLO). Open Forum Infect Dis 9. doi:10.1093/ofid/ofac492.646

[7] Hope WW, McEntee L, Livermore J, Whalley S, Johnson A, Farrington N, Kolamunnage-Dona R, Schwartz J, Kennedy A, Law D, Birch M, Rex JH. 2017. Pharmacodynamics of the orotomides against Aspergillus fumigatus: new opportunities for treatment of multidrug-resistant fungal disease. mBio 8:1–17. doi:10.1128/mBio.01157-17 PMC5565967 28830945

[8] Maertens JA, Thompson GR 3rd, Spec A, Donovan FM, Hammond SP, Bruns AHW, Rahav G, Shoham S, Johnson R, Rijnders B, et al.. 2025. Olorofim for the treatment of invasive fungal diseases in patients with few or no therapeutic options: a single-arm, open-label, phase 2b study. Lancet Infect Dis 25:1177–1188. doi:10.1016/S1473-3099(25)00224-5 40541222

[9] European Medicines Agency. 2012. Guideline on the investigation of drug interactions (CPMP/EWP/560/95/Rev. 1 Corr. 2*). Available from: https://wwwemaeuropaeu/en/documents/scientific-guideline/guideline-investigation-drug-interactions-revision-1_enpdf

[10] International Council for Harmonisation of Technical Requirements for Pharmaceuticals for Human Use (ICH). 2024. Drug interaction studies M12. Available from: https://databaseichorg/sites/default/files/ICH_M12_Step4_Guideline_2024_0521_0pdf

[11] Yimaz FF, Kennedy T, Allen G, Steiner J, Oliver J, Birch M, Sibley G, Law D. 2017. An open-label study in healthy volunteers to evaluate the potential for cytochrome P450 3A4 inhibition by F901318 using oral midazolam as a probe. 27th European Congress of Clinical Microbiology and Infectious Diseases. Vienna, Austria

[12] Cornelissen K, Rex JH, Allen G, Steiner J, Kennedy T. 2021. An open-label study in healthy volunteers to evaluate the effect of fluconazole upon the pharmacokinetics of olorofim. 31st European Congress of Clinical Microbiology and Infectious Diseases (Online).

[13] Cornelissen K, Newell P, Rex JH, Puri A. 2021. An open-label study in healthy volunteers to evaluate the effect of itraconazole and rifampicin upon the pharmacokinetics of a single oral dose of olorofim. 31st European Congress of Clinical Microbiology and Infectious Diseases (Online).

[14] Groll AH, Townsend R, Desai A, Azie N, Jones M, Engelhardt M, Schmitt-Hoffman A-H, Brüggemann RJM. 2017. Drug-drug interactions between triazole antifungal agents used to treat invasive aspergillosis and immunosuppressants metabolized by cytochrome P450 3A4. Transpl Infect Dis 19. doi:10.1111/tid.12751 28722255

[15] Brüggemann RJM, Alffenaar J-W, Blijlevens NMA, Billaud EM, Kosterink JGW, Verweij PE, Burger DM. 2009. Clinical relevance of the pharmacokinetic interactions of azole antifungal drugs with other coadministered agents. Clin Infect Dis 48:1441–1458. doi:10.1086/598327 19361301

[16] Cornelissen K, Zinzi D, Rex JH, Galbraith H, Neutel J, Phase MTA, Single-dose I. 2013. Phase I, single-dose, parallel group study to assess the pharmacokinetics of olorofim in subjects with hepatic impairment. 11th Trends in Medical Mycology (TIMM-11).

[17] Lee S, Kim B-H, Nam W-S, Yoon SH, Cho J-Y, Shin S-G, Jang I-J, Yu K-S. 2012. Effect of CYP2C19 polymorphism on the pharmacokinetics of voriconazole after single and multiple doses in healthy volunteers. J Clin Pharmacol 52:195–203. doi:10.1177/0091270010395510 21383338

[18] VFEND US Prescribing Information (USPI); VFEND (voriconazole) i.v., tablets and suspension label. 2025. https://www.accessdata.fda.gov/drugsatfda_docs/label/2010/021266s032lbl.pdf.

[19] Owusu Obeng A, Egelund EF, Alsultan A, Peloquin CA, Johnson JA. 2014. CYP2C19 polymorphisms and therapeutic drug monitoring of voriconazole: are we ready for clinical implementation of pharmacogenomics? Pharmacotherapy 34:703–718. doi:10.1002/phar.1400 24510446 PMC4082739

[20] Czyrski A, Resztak M, Świderski P, Brylak J, Główka FK. 2021. The overview on the pharmacokinetic and pharmacodynamic interactions of triazoles. Pharmaceutics 13:1961. doi:10.3390/pharmaceutics13111961 34834376 PMC8620887

[21] Miceli MH, Kauffman CA. 2015. Isavuconazole: a new broad-spectrum triazole antifungal agent. Clin Infect Dis 61:1558–1565. doi:10.1093/cid/civ571 26179012

[22] Saad AH, DePestel DD, Carver PL. 2006. Factors influencing the magnitude and clinical significance of drug interactions between azole antifungals and select immunosuppressants. Pharmacotherapy 26:1730–1744. doi:10.1592/phco.26.12.1730 17125435

[23] Livak KJ, Schmittgen TD. 2001. Analysis of relative gene expression data using real-time quantitative PCR and the 2^−ΔΔC_T_^ method. Methods 25:402–408. doi:10.1006/meth.2001.1262 11846609

[24] Wagner C, Pan Y, Hsu V, Grillo JA, Zhang L, Reynolds KS, Sinha V, Zhao P. 2015. Predicting the effect of cytochrome P450 inhibitors on substrate drugs: analysis of physiologically based pharmacokinetic modeling submissions to the US Food and Drug Administration. Clin Pharmacokinet 54:117–127. doi:10.1007/s40262-014-0188-4 25260695

[25] Quinney SK, Zhang X, Lucksiri A, Gorski JC, Li L, Hall SD. 2010. Physiologically based pharmacokinetic model of mechanism-based inhibition of CYP3A by clarithromycin. Drug Metab Dispos 38:241–248. doi:10.1124/dmd.109.028746 19884323 PMC2812061

