# Supplementary material for: Evaluation of the cytochrome P450-mediated drug interaction profile of olorofim
Source: Antimicrob Agents Chemother. 2026 Apr 20;70(6):e01745-25. doi: 10.1128/aac.01745-25 (PMC13231911; doi:10.1128/aac.01745-25)
Supplement: Supplemental material — Tables S1 to S4. [file aac.01745-25-s0001.docx]

**Table S1: Summary of olorofim CYP phenotyping data using human liver microsomes with specific CYP inhibitors.**

| **Isoform** | **CYP Inhibitor** | **CYP Positive control** | **Olorofim CL_int_ (µL/min/mg protein) ^1^** | **Olorofim mean CL_int_ ratio ^2^ (%)** |
| --- | --- | --- | --- | --- |
| CYP1A2 | alpha-naphthoflavone | ethoxycoumarin | 63.8 | 115 |
| CYP2B6 | ticlopidine | efavirenz | 50.4 | 90.8 |
| CYP2C8 | montelukast | amodiaquine | 43.8 | 78.9 |
| CYP2C9 | sulphaphenazole | diclofenac | 38.7 | 69.7 |
| CYP2C19 | N-3-benzyl-phenobarbital | diazepam | 47.9 | 86.3 |
| CYP2D6 | quinidine | dextromethorphan | 49.1 | 88.5 |
| CYP3A4 | ketoconazole | testosterone | 9.84 | 17.7 |
| No inhibitor | | | 55.5 | N/A |
| ^1^ determined in microsomes in the presence of specific inhibitor for each isoform  ^2^ ratio of Cl_int_ with inhibitor to Cl_int_ without inhibitor  N/A: Not applicable | | | | |

**Table S2: Summary of demographics for the clinical studies assessing olorofim’s DDI profile**

| Study number  (Clintrials.gov number) | Population | Gender  (N) | Mean Age [range] (years) | Mean BMI [range] (kg/m2) |
| --- | --- | --- | --- | --- |
| F901318-01-04-15  (NCT02680808) | Healthy volunteers | Male: 20 [100%] | 29.6  [18 – 44] | 24.8  [20.3 – 29.9] |
| F901318-01-05-15  (NCT02730442) | Healthy volunteers | Male: 30 [94%]  Female: 2 [6%] | 32.1  [20 – 45] | 25.7  [21.1 – 32.25] |
| F901318-01-06-16^1^  (NCT02737371) | Healthy volunteers | Male: 3 [43%]  Female: 4 (57%] | 31.3  [24 – 37] | 26.2  [22.7 – 30.2] |
| F901318-01-15  [itraconazole DDI]  (NCT04171739) | Healthy volunteers | Male: 10 [83%]  Female: 2 [17%] | 39.3  [23-54] | 24.1  [18.7 – 28] |
| F901318-01-15  [rifampicin DDI]  (NCT04171739) | Healthy volunteers | Male: 11 [92%]  Female: 1 [8%] | 33.5  [19 - 55] | 24.7  [22.1 – 30] |
| Phase 2b (NCT03583164) | IFD patients | Male: 124 [61%]  Female: 79 [39%] | 53.1  [18 – 90.4] | 24  [14.2 – 43.2] |

^1^ Control group for fluconazole DDI assessment

**Table S3: PKPB model input parameters**

| **PARAMETER** | **Value** | **Data source** |
| --- | --- | --- |
| **Physicochemical and Binding Parameters** | | |
| Molecular Weight (g/mol) | 498.55 | CMC data |
| Log P | 3.95 | CMC data |
| Compound type | Monoprotic Base | CMC data |
| pKa | 3.14 | CMC data |
| Blood:Plasma | 0.578 | *In vitro* studies |
| fu | 0.002 | *In vitro* studies |
| Main binding protein | AGP | Assumed |
| **ADAM Absorption Model** |  |  |
| fugut | 0.002 | Equal to fu |
| Caco-2 Papp (x10-6 cm/s) pH 6.5:7.4 | 10.7 | In vitro study data |
| Peff,man (pred) (x10-4 cm/s) | 1.64 | Predicted |
| Formulation type | Solution | Dissolution assumed to be non rate-limiting |
| **Distribution Model – Minimal PBPK Model** | | |
| V_ss_ (L/kg) | 3.08 | Clinical data (study F901318-01-01-14) |
| K_in_ (1/h) | 0.443 | Estimated from study F901318-01-01-14 |
| K_out_ (1/h) | 0.107 | Estimated from study F901318-01-01-14 |
| V_SAC_ (L/kg) | 2.38 | Estimated from study F901318-01-01-14 |
| **Elimination Parameters** | | |
| CYP3A4 CLint (μl/min/pmol) | 6.52 | Retrograde model; fmCYP3A4 0.717 |
| CYP2C9 CLint (μl/min/pmol) | 3.45 | Retrograde model; fmCYP2C9 0.186 |
| CYP2C8 CLint (μl/min/pmol) | 4.97 | Retrograde model; fmCYP2C8 0.0772 |
| CYP2C19 CLint (μl/min/pmol) | 3.40 | Retrograde model; fmCYP2C19 0.0124 |
| CYP2D6 CLint (μl/min/pmol) | 1.13 | Retrograde model; fmCYP2D6 0.00755 |
| Additional HLM CLint (μL/min/mg) | 0.116 | Retrograde model |
| CLR (L/h) | 0 | Clinical data (studies F901319 01-01-14 and F901318-01-09) |
| **Interaction Parameters** | | |
| CYP3A4 kinact (h-1) | 5.325 | Optimised (DDI Study F901318-01-04-15) |
| CYP3A4 KI,u (μM) | 3.26  (Corrected for predicted fumic of 0.823) | *In vitro* studies |
| CYP2D6 kinact (h-1)  Only applied in application simulation with a CYP2D6 substrate | 3.28 | *In vitro* studies |
| CYP2D6 KI,u (μM)  Only applied in application simulation with a CYP2D6 substrate | 2.91  (Corrected for predicted fumic of 0.823) | *In vitro* studies |
| CYP1A2 Ind_max_ | 2.16 | *In vitro* studies |
| CYP2B6 Ind_max_ | 1.99 | *In vitro* studies |

**Table S4: Key PKPB model verification results**

| **Study** | **Output** | **AUC**  **(µg·h/mL)** | **C_max_**  **(µg/mL)** |
| --- | --- | --- | --- |
| Single oral dose (150 mg olorofim) | Simulated  (trial range) | 13.3 ^a^  (11.6 – 15.8) | 1.29  (1.07 – 1.54) |
|  | Observed | 14.7 ^a^ | 1.11 |
|  | S/O  (trial range) | 0.91  (0.79 – 1.08) | 1.16  (0.96 – 1.39) |
| Multiple oral dose  (90 mg BID olorofim; Day 10) | Simulated  (trial range) | 11.3 ^b^  (7.52 – 14.58) | 1.62  (1.06 – 2.26) |
|  | Observed | 11.9 ^b^ | 2.21 |
|  | S/O  (trial range) | 0.95  (0.63 – 1.24) | 0.73  (0.48 – 1.02) |
| **DDI study** | **Output** | **GMR for AUC** | **GMR for C_max_** |
| single oral dose, 60 mg olorofim in presence and absence of itraconazole | Simulated  (trial range) | 2.62  (2.44 – 2.90) | 1.38  (1.25 – 1.55) |
|  | Observed | 2.40 | 1.52 |
|  | S/O  (trial range) | 1.09  (1.02 – 2.66) | 0.91  (0.82 – 1.02) |
| single oral dose, 120 mg olorofim in presence and absence of rifampicin | Simulated  (trial range) | 0.24  (0.19 – 0.26) | 0.51  (0.40 – 0.57) |
|  | Observed | 0.26 | 0.56 |
|  | S/O  (trial range) | 0.92  (0.73 – 1.00) | 0.91  (0.71 – 1.02) |
| S/O: simulated / observed data; GMR: Geometric mean ratio  ^a^ AUC_0-120 h_ ^b^ AUC_0-12 h_ | | | |
